# Factors associated with using the internet for medical information based on the doctor-patient trust model: a cross-sectional study

**DOI:** 10.1186/s12913-021-07283-6

**Published:** 2021-11-24

**Authors:** Yang Fu, Tianwei Tang, Junhao Long, Bohuai Lin, Jiayue Li, Guohong Quan, Hanwen Yang, Chongbang Zhao, Mei Yin, Lei Shi

**Affiliations:** 1 School of Humanities, Harbin Medical University, Harbin, Heilongjiang China; 2 The Second Affiliated Hospital of Harbin Medical University, Harbin, China; 3 The Third Affiliated Hospital of Sun Yat-sen University, Guangzhou, China; 4 School of Health Management, Southern Medical University, Guangzhou, China

**Keywords:** Medical information, Internet medical, Trust, Behavioural intentions, Patient

## Abstract

**Background:**

Internet medical care has been advancing steadily, especially during the coronavirus disease 2019 pandemic, the development momentum of Internet medical care in China is more vigorous. This study aimed to explore the factors associated with using the Internet for medical information, to examine the popularisation and implementation of Internet medical treatment and feasible strategies, and promote the further development of Internet medical treatment.

**Methods:**

A cross-sectional study was conducted on 408 medical patients who had used online medical services. The one-way analysis of variance or independent samples t-test was used to compare the differences in the influence of demographic characteristics on behavioural intentions of different people seeking medical care. Pearson’s correlation was used to evaluate the correlation between different measurement variables. A mediation regression analysis was used to explore the mediating role of trust in Internet medical care.

**Results:**

The difference in the influence of Internet medical use frequency on the behavioural intention of different participants was statistically significant (F = 3.311, *P* = 0.038). Among the influencing factors, personal trust propensity (*r* = 0.387, *P* < 0.01), website credibility (*r* = 0.662, *P* < 0.01), hospital credibility (*r* = 0.629, *P* < 0.01), doctor’s credibility (*r* = 0.746, *P* < 0.01), and online patient trust (*r* = 0.874, *P* < 0.01) were positively correlated with patients’ behavioural intentions. In the analysis of intermediary factors, the total effect of the credibility of the diagnosis and treatment website on the behavioural intention of patients was 0.344. The total effect of the credibility of the diagnosis and treatment hospital on the behavioural intention of patients was 0.312; the total effect of the service doctor’s credibility on the patient’s behavioural intention was 0.385; the total effect of the personal trust tendency on the patient’s behavioural intention was 0.296.

**Conclusions:**

This study found defects in various factors that produce distrust in Internet medical treatment. It also reveals the positive effect of trust factors on the development and implementation of Internet medical treatment and provides some ideas for improving the use of Internet medical treatment by the masses.

## Background

With the development of society and economics, and the ageing of the population, medical and management practitioners are discussing how people can use technology and the Internet to improve medical services [1–3]. Supplementary to the existing traditional medical model, Internet medical treatment brings convenience to patients with unique online medical and health information provision, alleviates the problem of ‘difficulty in seeing a doctor’, and facilitates the harmonious doctor-patient relationship more effectively [4, 5].

Internet medical services started relatively early, such as Epocrates and ZocDoc, and established mature Internet medical business models by charging pharmaceutical companies, doctors, and patients, forming a huge Internet medical system. For example, Miranda et al.’s study found that the use of electronic health records and Epocrates provided accurate prescription information for 93.1 and 89.4% of drugs, respectively, in their research on Internet medical mobile applications [6]. Internet medical care has been advancing steadily, especially during the coronavirus disease 2019 (COVID-19) pandemic, the development momentum of Internet medical care in China is more vigorous [7]. The report of 2020 Internet Hospital showed that ‘Ping A Good Doctor’ platform and ‘Dingxiangyuan Epidemic Map’ had more than 100 million visits and growth was rapid [8]. The rapid development of the pandemic leads to an increase in the demand for medical consultation. Kakanip interviewed hundreds of thousands of patients in California hospitals, and found that 0.5% of the patients had used Internet medical treatment before the pandemic, while the proportion increased to 41.2% during the pandemic [9]. Therefore, we can see the great potential of Internet medical treatment in public health emergencies such as the COVID-19 pandemic.

How can the development of Internet medical care be accelerated? Previous studies have shown that trust is an important factor affecting the use of Internet medical care. Akter’s study showed that perceived trust has an impact on the continued use of mHealth under the expectation confirmation model [10]. Krebs also found high rates of distrust of data security among consumers who have not used a health app [11]. Trust can be viewed as an antecedent that may create a positive attitude toward behavioural intentions [12]. Bart and Shankar found that trust is a key element that affects behavioural intention [13]. Moreover, Hong also indicated that trust is a predictor for the continued use intention of online healthcare services in China [14].

### Related concepts of internet medical care

There is still no clear distinction and definition of the concept of Internet medical care. Meng proposed that Internet medical service is the general term describing new medical and health service formed by the deep integration of the Internet as the carrier (act as a platform, such as various health websites) and information technology (including mobile communication technology, cloud computing, Internet of Things, big data, etc.) with traditional medical and health services [15]. The Health Resources and Services Administration defines telehealth as the use of electronic information and telecommunication technologies, such as video conferencing, to support remote clinical care, patients and health-related professional education, public health, and health management [16]. The Centres for Medicare and Medicaid Services believe that telemedicine is a two-way real-time interaction between doctors and patients located far apart from each other to improve patients’ health [17] .‘Internet + medical services’ is a new concept to promote the implementation of the Healthy China strategy and promote the allocation of medical resources in the new era [18]. In 2018, the National Health Commission and the National Administration of Traditional Chinese Medicine jointly issued the ‘Notice on the issuance of three documents including the Administrative Measures for Internet Diagnosis and Treatment (Trial)’, which divided ‘Internet + Medical Services’ into telemedicine, Internet diagnosis and treatment activities, and Internet hospitals according to the personnel and service modes used [18]. The Internet medical treatment targeted in this study mainly refers to the Internet + medical service, which refers to the medical behaviour that people use the Internet to seek help from medical personnel, including consultation, disease diagnosis and treatment, return visits, and access to health information [18].

### Related concepts of doctor-patient trust

Trust is the focus of research in fields such as psychology and sociology. Weigert and Lewis divided trust into cognitive trust and emotional trust, and believed that trust arises from people’s social interactions and there are considerable differences in the connotation and manifestations of trust among different social groups, although the theories and models of trust vary greatly in various fields, according to previous studies on trust by scholars, trust can be summarised as ‘a psychological state comprising the intention to accept vulnerability based upon positive expectations of the intentions or behaviour of another’ [19].

Trust is an important foundation for the establishment and maintenance of interpersonal relationships, and the doctor-patient relationship is one of many social relationships in which trust plays a particularly important role [20]. The doctor-patient trust refers to a contractual relationship established by both sides in the process of interaction [20]. This contractual relationship is often manifested as patients entrusting their lives and health to doctors, and doctors carrying the trust of patients. Doctor-patient trust can improve patient compliance, reduce the contradiction between doctors and patients, and promote the harmonious coexistence of doctors and patients [20]. In the traditional medical model, patients are in a relatively weak position due to the information asymmetry between doctors and patients. Therefore, this study focuses on patient trust.

### Impact of doctor-patient trust on internet healthcare

Compared with the traditional medical model in the past, the introduction of Internet medical services has brought about risks such as information leakage, which challenges the trust between doctors and patients even more. Kim [21], Sbaffi, and Rowley [22], when evaluating influencing factors of patients’ trust in the health information network, believed that individual characteristics of patients and website-related factors were correlated with patient trust. Peng found that patients’ perceived usefulness of online medical information and doctor services can promote doctor-patient interaction and enhance doctor-patient trust [23]. Dongwei believed that there is a correlation between patients’ trust in medical staff and patients’ risk perception during treatment [24]. In a study of doctor-patient trust in offline medical treatment, Li et al. believed that hospital management factors such as doctors’ skills and attitudes, hospital regulations and treatment procedures, as well as policy factors such as the medical insurance system and hospital compensation mechanism would affect doctor-patient trust [25]. At present, most research on online trust is concentrated in the field of e-commerce, and most of the theoretical models of trust are also applied. There is still a lack of a more comprehensive analysis of the composition of doctor-patient trust between consumers and service providers in the market. Therefore, this study will analyse the influence of individuals, websites, hospitals, and doctors, and verify the hypothesis using a questionnaire survey.

### Research hypothesis

The subject of this research is centred on the influence of patient trust factors on the use of Internet medical treatment. The purpose of this study is to explore the relationship between trust factors as intermediary factors among various factors and patients’ behavioural intentions. Combined with the established theoretical basis and the analysis of the questionnaire results, a theoretical model was established, and data analysis was carried out to test the relationship between trust factors and patient behavioural intentions. Second, under the current social development, it is hoped that the positive influence of trust factors on the behavioural intention of patients can be confirmed to provide ideas for promoting the development of Internet medical treatment.

We make the following assumptions:

Hypothesis A: Personal trust tendencies where the tendency is to trust positively influences patients’ behaviour intentions in Internet medical care..

Hypothesis B: Personal trust tendencies where the tendency is to distrust negatively influences patients’ behaviour intentions in Internet medical care.

Hypothesis C: The credibility of the platform, medical hospitals and serving doctors positively affects the behavioural intention of patients in Internet medical care.”

## Methods

### Samples and participants

From 15 November 2020 to 9 January 2021, we conducted a cross-sectional survey on the trust of doctors and patients in online healthcare services. Purposive sampling method was used to survey 408 medical patients who had used online medical treatment in three tertiary hospitals in Harbin, Heilongjiang Province. Sample extraction process: First, three public tertiary hospitals in Harbin were randomly selected. Second, with the assistance of the hospital’s medical department, the outpatient population was surveyed. On average, each hospital surveyed 150 people who had used online medical treatment. Before the survey had officially started, we conducted a preliminary survey and distributed 30 questionnaires (these data were not included in the final analysis). Increased technology supports low-budget online surveys as a common method for collecting information [26]. At the same time, considering that relative social distance should be maintained during the pandemic, electronic questionnaires (ID: 97500543) are mainly used for investigation. A total of 408 questionnaires were sent out. Exclusion criteria was filling the questionnaire in a very short amount of time (< 300 s). Finally, 336 valid questionnaires were obtained, and the questionnaire’s efficiency was 82.35%. Eligibility criteria were voluntary participation and use of online medical services in the past year.

### Measurements

The questionnaire content used in this study was divided into two sections:

1. The first section was the basic situation survey form, that is, demographic data, which mainly includes gender, age, educational background, location, personal trust tendencies, chronic diseases, etc.
2. The second section was a survey of doctor-patient trust in online medical services, which is divided into 5 dimensions and 35 items. The various dimensions and items are summarised as follows:

First, the ‘personal trust tendency’ dimension comprised four items and was located in the first part of the basic situation questionnaire. This dimension was revised and developed based on Hoffmann et al.’s related research on online trust to measure personal trust tendency [27]. Each item of this dimension is rated on a 5-point Likert scale to express the degree of conformity between the respondent and item options with 1 being ‘very consistent’, 2 ‘conforms’, 3 ‘fair’, 4 ‘non-conformity’, and 5 ‘very non-conformance’. The lower the score, the more likely the interviewee was to trust others, and the higher the score, the more difficult it was for the interviewee to trust others. The McDonald’s ω value of the scale was 0.825 in this study.

Second, the ‘platform credibility’ dimension was based on the research of Harris et al. [28]. on website credibility, and is mainly used to measure the degree of recognition of online medical network platforms by patients. It comprises six items, each of which are rated on a 5-point Likert scale to express the degree of conformity between the respondent and the item options with 1 being ‘very consistent’ and 5 ‘very inconsistent’. The lower the score, the higher the credibility of the platform website considered by the patient, and vice versa. The McDonald’s ω value of the scale was 0.864 in the present study.

Third, the ‘hospital credibility’ dimension is based on the research of Ractham et al. [29]. on patients’ intentions for medical tourism. It was mainly used to assess the degree of recognition of patients in hospitals where online medical care is located. This dimension comprises six questions, each of which were rated on a 5-point Likert scale with 1 being ‘very consistent’ and 5 ‘very non-compliant’. The lower the score, the more credible and the higher the degree of recognition the patient believes the hospital to receive. The higher the score, the lower the credibility of the hospital and poorer service capabilities. The McDonald’s ω value of the scale was 0.883 in the present study.

Fourth, the ‘doctor’s credibility’ dimension was derived from the Anderson et al’s research [30]. on the development of the doctor trust scale. The dimension is used to measure the degree of trust of the patient’s online doctors. This dimension comprises six items, each of which are rated on a 5-point Likert scale, with 1 being ‘very consistent’ and 5 ‘very inconsistent’. The lower the score, the higher patients’ recognition of and satisfaction with the doctor, and the more they trust him. The McDonald’s ω value of the scale was 0.874 in the present study.

Fifth, the ‘trust degree of online medical treatment’ dimension is based on the research of Shankar et al. [31] and Liu et al. [32] on online trust and privacy trust, respectively. This dimension comprises seven items in two parts, of which four items are regarding trust. The lower the score, the more trust the patient has in the form of online medical treatment and the better their experience of diagnosis and treatment. In contrast, the worse the experience of the patient in completing the online medical form, the more distrustful the patient. Regarding the three items addressing behaviour intention, a lower score indicates more optimism about online medical care and higher willingness to use it again or recommend it to others. The higher the score, the less willing the patient is to use online medical care and the more negative impression of online medical treatment. All items in this dimension are rated on a 5-point Likert scale with 1 being ‘very consistent’ and 5 ‘very non-compliant’. In this scale, the omega value of the online trust part was 0.809, and the omega value of the behaviour intention part was 0.725.

### Data analysis

IBM SPSS25.0 was used for data analysis. Multiple interpolation methods were used to deal with incomplete data in this analysis. P-P diagram and Kolmogorov-Smirnov tests were used to verify the normal distribution of continuous variables. Descriptive statistics obtained include sample size (N), percentage(%), and Mean (SD). We used a one-way analysis of variance or independent samples t-test to compare the differences in the influence of demographic characteristics on behavioural intentions of different people seeking medical care. Pearson’s correlation was used to evaluate the correlation between different measurement variables. We chose Process v3.4.1 [33], a software package for mediation analysis developed by Hayes, and controlled the frequency of use of health websites, the reliability of diagnosis and treatment websites, the reliability of diagnosis and treatment hospitals, and the reliability of service doctors. Personal trust tendency is an independent variable (X), trust in Internet medical care is used as an intermediary factor (M), and patient behavioral intention is a dependent variable (Y). Using model 4, the sample size is set to 5000 to get the final Data analysis results of intermediary factors. Statistical significance was defined as a two-tailed *P* < 0.05.

## Results

### Demographic characteristics of the respondents

Among the 336 respondents, the majority were women (56.8%). Most were in the age group under 30 (33.6%) and 41–50 (34.8%) years old. The area was mainly urban, accounting for 87.2% (Table 1).

**Table 1:** Demographic characteristics of the respondents (*N*=336)

Demographic variables		n	%
Gender	Male	145	43.2
	Female	191	56.8
Age group	≤30	113	33.6
	31-40	46	13.7
	41-50	117	34.8
	≥51	60	17.9
Educational level	Junior college or below	145	43.2
	Bachelor degree or above	191	56.8
Area	City	293	87.2
	Village	43	12.8
Suffering from chronic disease	Yes	67	19.9
	No	269	80.1
Usage frequency	Once or more times a week	14	4.2
	Once a month	23	6.8
	Once every two months or more	299	89.0

### The influence of the frequency of internet medical use on behavioural intention among different participants

The results in Table 2 show that the differences in the frequency of Internet medical use of different participants on behavioural intentions were statistically significant (*p* = 0.038). There was no statistically significant difference in the influence of different participants’ gender, age, educational background, location, and chronic disease on behavioural intention (*p* > 0.05).

**Table 2:** The influence of the frequency of internet medical use onbehavioral intention among different participants

Variables		Behavior intention		***F/t***	***p***
		***Mean***	***SD***		
Gender	Male	7.07	2.49	1.337	0.182
	Female	6.71	2.37		
Age group	≤30	7.22	2.31	1.688	0.169
	31-40	6.87	2.55		
	41-50	6.50	2.39		
	≥51	6.90	2.57		
Educational level	Junior college or below	6.83	2.54	-0.253	0.800
	Bachelor degree or above	6.90	2.35		
Location	City	6.78	2.41	-1.807	0.072
	Village	7.49	2.46		
Suffering from chronic disease	Yes	6.88	2.43	0.055	0.956
	No	6.86	2.43		
Usage frequency	Once or more times a week	5.29	1.82	3.311	0.038
	Once a month	6.65	2.39		
	Once every two months or more	6.96	2.44		

### Correlation analysis among different variables

The average personal factor was 9.274, the average of the website was 13.866, the average of the hospital was 12.819, and the average of the doctor was 13.560. The average value of personal factors in the survey was the lowest, indicating the highest level of personal trust tendencies. The results in Table 3 showed that personal trust tendency (*r* = 0.387, *p* < 0.01), website credibility (*r* = 0.662, *p* < 0.01), hospital credibility (*r* = 0.629, *p*<0.01), doctor’s credibility (*r* = 0.746, *p* < 0.01), and online patient trust (*r* = 0.874, *p* < 0.01) were positively correlated with patients’ behavioural intentions.

**Table 3:** Pearson correlation analysis among different variables

Variables	1	2	3	4	5	6	7
1.Frequency of use of online health websites	1						
2.Personal trust tendency	0.047	1					
3.Website credibility	0.137*	0.425**	1				
4.Hospital credibility	0.108*	0.391**	0.774**	1			
5.Doctor's credibility	0.151**	0.405**	0.739**	0.803**	1		
6.Online patient trust	0.157**	0.405**	0.690**	0.640**	0.776**	1	
7.Patient's behavior tendency	0.131*	0.387**	0.662**	0.629**	0.746**	0.874**	1

**P<0.01

*P<0.05

### Analysis of mediating role

**Fig. 1:**
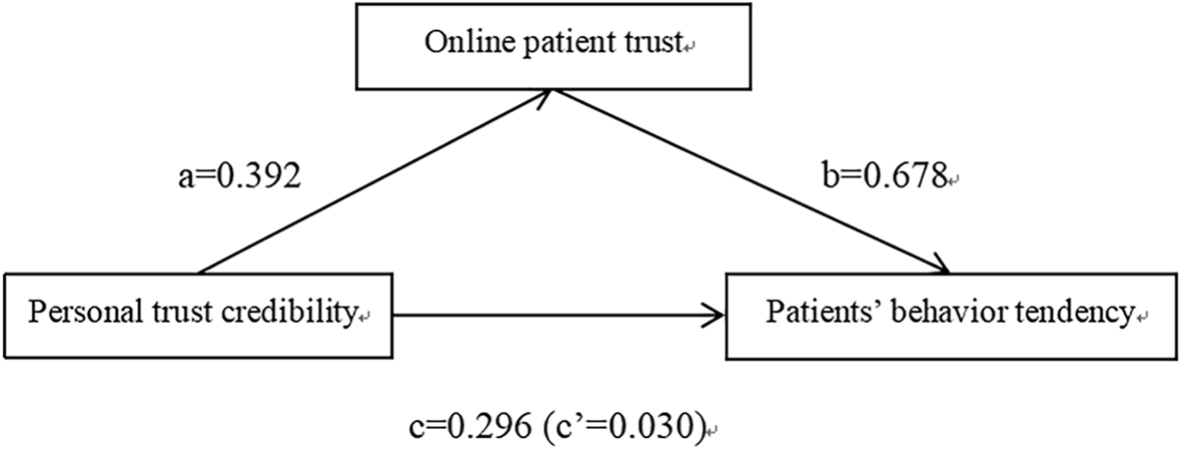
The mediating role of online patient trust in explaining the relation between the personal trust credibility and patients’ behavior tendency (path 1 in Table 4). *N* = 336; controlled for gender,age group,education level,area,suffering from chronic disease; a = direct effect of personal trust credibility on mediator; b = direct effect of mediator on patient’s behavior tendency;c = total effect of personal trust credibility on patient’s behavior tendency; c’ = direct effect of personal trust credibility on patient’s behavior tendency

Table 4 shows: 1) The overall influence of website reputation on patients’ behavioral intentions is significant, which is 0.344; the indirect influence on Internet medical trust is 0.285; The direct influence of website reputation is significant at 0.058, which indicates that the influence of website reputation on the behavioral intention of patients in the intermediary role has a significant effect on online medical services. 2) The overall effect of hospital credibility on patients’ behavioural intentions was significant at 0.312; indirect effect on Internet medical trust was significant at 0.253; and direct effect of the credibility of the medical hospital was significant at 0.059, indicating that the degree of trust in online medical treatment plays a part in the mediating role in the influence of the credibility of the medical hospital on the behaviour intention of patients. 3) The total effect of the doctor’s credibility on the behaviour intention of patients is significant at 0.385; indirect effect on the trust level of Internet medical treatment was significant at 0.296; and direct effect of the doctor’s credibility was significant at 0.088, indicating that the degree of trust in online medical treatment plays a mediating role in the influence of the doctor’s credibility on the behaviour intention of patients. 4) The overall effect of personal trust tendency on patient’s behaviour intention was significant at 0.296; indirect effect on Internet medical trust was significant at 0.266; and direct effect of personal trust tendency was not significant at 0.030, indicating that the degree of trust in online medical treatment plays a complete mediating role in the influence of personal trust tendency on patient’s behaviour intention. The mediating role of online patient trust in explaining the relation between the personal trust credibility and patients behavior tendency (Fig. 1).

**Table 4:** Results of mediation analyses

Paths	a	b	c’	a*b	95% CI of a*b	c	***R***^**2**^
1.Personal trust tendency→Online patient trust→Patient's behavior tendency	0.392	0.678	0.030	0.266	(0.185, 0.345)	0.296	0.765
2.Hospital credibility→Online patient trust→Patient's behavior tendency	0.402	0.630	0.059	0.253	(0.210, 0.295)	0.312	0.772
3.Doctor's credibility→Online patient trust→Patient's behavior tendency	0.507	0.585	0.088	0.296	(0.251, 0.345)	0.385	0.557
4.Website credibility→Online patient trust→Patient’s behavior tendency	0.454	0.629	0.058	0.285	(0.239, 0.335)	0.344	0.770

## Discussion

The focus of this study is the impact of patient trust factors on Internet medical use. From the relationship between personal trust, website, hospital, doctor, Internet doctor-patient trust and behavior willingness, it has successfully verified the intermediary role of Internet doctor-patient trust. Our findings highlight the current deficiencies of the Internet, providing an effective and targeted strategic basis to popularize and promote the development of Internet medical treatment.

### Credibility of hospital

Patient trust plays an intermediary role between the credibility of hospitals and patients’ behaviour intentions, which were similar to results of Ju and colleage [34]. This may be because the hospitals selected by most Internet platforms are among the top hospitals in the country, with no big difference for patients. To improve credibility of hospitals, hospitals should pay more attention to the construction of a network platform and media publicity, so patients can have a deeper understanding of each hospital and know the strong departments of these hospital, allowing them the best treatment approach.

### Credibility of doctors

Patient trust plays an intermediary role between doctors’ credibility and patients’ behavioural intentions, and has a significant impact on patients’ behavioural intentions; therefore, it can be used to improve doctors’ credibility as a starting point, continuously improving the strength and credibility of doctors to improve the quality of online medical services used by patients. According to a survey in China, among the physicians and hospitals selected by patients, 55.4% of physicians are chief physicians or associate chief physicians, and 98% of tertiary hospitals [34] and inpatients have significantly higher confidence in doctors’ skills and total trust than outpatients [35]. This is due to the urgency of outpatient visits, short visits, and insufficient understanding of doctors and hospitals, while inpatients have more contact with medical staff and the hospital environment and have a deeper understanding of the work of medical staff. In the Internet medical process, the communication process between doctors and patients is shorter, and it is more difficult to obtain the trust of patients if they cannot communicate face-to-face. To address the problem that Internet medical care cannot establish good doctor-patient communication, we can learn from Priya Nambisan’s related conclusions [36] :Doctors should improve their empathy to narrow the distance between them and their patients, so that patients can express their demands in a shorter time, thereby improving communication efficiency, and making patients trust their doctors more and providing The online medical service platform can provide regular service quality assessments for every doctor who provides services, and show them to patients to enhance their understanding and trust in doctors.

### Personal trust tendencies

Patient trust plays a complete intermediary role between personal trust tendencies and patient’s behavioural intention. Online patient trust is a positive factor for behavioural intentions. An increase in personal trust tendencies can increase the degree of trust in Internet medical care and have a positive effect. Therefore, while improving other influencing factors, it is necessary to improve the trust of patients and change their behavioural intentions. According to Table 2, the behavioural intention scores of respondents with low education levels were low. This may be related to a lack of health knowledge, insufficient knowledge of one’s own diseases and health, difficulty in understanding the treatment methods and conditions described by doctors, and communication difficulties with medical staff. According to a survey, 58.7% of medical staff said that patients with a higher education level were more likely to communicate [37]. Based on this, strengthening the publicity and education of patients and guiding patients to control their self-expectations will effectively improve the quality of online medical services.

### Credibility of website

Patient trust plays an intermediary role between credibility of a website and patients’ behavioural intentions. In Ju’s survey results [34], the role of the platform is the most important, especially for patients with chronic diseases who hope for quick responses regarding information about their illness. The speed of receiving feedback directly affects the user experience. In addition, the richer the evaluations of doctors provided on the website and the more detailed the treatment methods, the more helpful it is for patients to choose a doctor that suits them. The doctor’s evaluation will affect their choice at the first use, and the speed of feedback received is the most important factor affecting subsequent re-selection. Improving trust in the website plays a positive role in developing trust in Internet medical services; so we can increase the frequency of using online medical services by improving patients’ trust in the website. Improving trust in the website can be achieved by improving security, privacy protection, and ease of use, thereby improving patients’ trust in Internet medical treatment.

To this end, we propose the following suggestions: First, doctors should increase their response speed to improve patients’ perceptions of them and make them more willing to cooperate with their diagnosis and treatment processes on the website. Secondly, the website should allow patients to evaluate doctors after each consultation. This will allow doctors to treat patients more proactively and allow other patients to better choose doctors that suit them. Third, the hospital should increase its publicity, provide more standardized and unified training for doctors, make the entire consultation process more unified, and reflect the overall image of the hospital.

This study has several limitations: First, the questionnaire survey method is greatly affected by individual subjective factors. Although the effectiveness of the questionnaire is improved by screening out the answers with a short answering time, certain errors may still exist. Follow-up research can reduce errors by interviewing or adding screening conditions. Second, the scope of Internet medical treatment is broad, and our research is still lacking in specificity. In the future, scholars can conduct corresponding research on more detailed Internet medical categories such as Internet hospitals, diagnosis, and treatment activities. Third, this electronic questionnaire survey mainly concentrated on urban areas, and coverage in rural areas may not be sufficient. Follow-up research can focus on the investigation of rural patients and compare the differences in factors affecting the trust of urban and rural patients in Internet medical care. Finally, a fundamental pre-condition for any causal interpretation is that the (assumed) cause is before the (assumed) effect. Given that a mediation assumes three causal relationships one would need at least three points of measurement with a reasonable time-lag to have a powerful mediation analysis. This study is a cross-sectional study, which limits the discussion of the causal relationship between variables.

## Conclusions

This research refers to previous studies based on online trust theory and combines online and offline trust influencing factors. From the four perspectives of individuals, hospitals, doctors, and platforms, this article explores the influence of trust factors on patients’ behavioural intentions in Internet medical activities. The results show that increasing the credibility of websites, hospitals, and doctors can improve online patient trust.

The significance of this research lies in the fact that Internet medical care supplements offline medical care. With its unique advantages and value in epidemic prevention and control, its development and growth is an inevitable trend, and should be promoted and improved. This research can provide medical workers with ideas to promote the development of Internet medical care and provide reference strategies for the development of online medical and health services.

## Data Availability

The datasets and/or analyses from the current study will be available from the corresponding authors upon reasonable request.

## Abbreviation

COVID-19: Coronavirus disease 2019
